# Effects of diet, habitat, and phylogeny on the fecal microbiome of wild African savanna (*Loxodonta africana*) and forest elephants (*L. cyclotis*)

**DOI:** 10.1002/ece3.6305

**Published:** 2020-05-18

**Authors:** Kris Budd, Joe C. Gunn, Tabitha Finch, Katy Klymus, Noah Sitati, Lori S. Eggert

**Affiliations:** ^1^ Division of Biological Sciences University of Missouri Columbia MO USA; ^2^ Vermont Genetics Network University of Vermont Burlington VT USA; ^3^ Columbia Environmental Research Center United States Geological Survey Columbia MO USA; ^4^ World Wide Fund for Nature Dar es Salaam Tanzania

**Keywords:** 16S gene, crop‐raiding, frugivorous diet, gut microbiome, herbivore

## Abstract

The gut microbiome, or the community of microorganisms inhabiting the digestive tract, is often unique to its symbiont and, in many animal taxa, is highly influenced by host phylogeny and diet. In this study, we characterized the gut microbiome of the African savanna elephant (*Loxodonta africana*) and the African forest elephant (*Loxodonta cyclotis*), sister taxa separated by 2.6–5.6 million years of independent evolution. We examined the effect of host phylogeny on microbiome composition. Additionally, we examined the influence of habitat types (forest versus savanna) and diet types (crop‐raiding versus noncrop‐raiding) on the microbiome within *L. africana*. We found 58 bacterial orders, representing 16 phyla, across all African elephant samples. The most common phyla were Firmicutes, Proteobacteria, and Bacteroidetes. The microbiome of *L. africana* was dominated by Firmicutes, similar to other hindgut fermenters, while the microbiome of *L. cyclotis* was dominated by Proteobacteria, similar to more frugivorous species. Alpha diversity did not differ across species, habitat type, or diet, but beta diversity indicated that microbial communities differed significantly among species, diet types, and habitat types. Based on predicted KEGG metabolic pathways, we also found significant differences between species, but not habitat or diet, in amino acid metabolism, energy metabolism, and metabolism of terpenoids and polyketides. Understanding the digestive capabilities of these elephant species could aid in their captive management and ultimately their conservation.

## Graphical abstract

The gut microbiome, or the community of microorganisms inhabiting the digestive tract, is often unique to its symbiont and, in many animal taxa, is highly influenced by host phylogeny and diet. We studied the microbiome African savanna and forest elephants, and the influence of habitat types (forest patches versus savanna) and diet types (crop‐raiding versus noncrop‐raiding) on the microbiome of savanna elephants. We found that alpha diversity did not differ across species, habitat type, or diet, but beta diversity differed significantly among species, diet types, and habitat types, and there are significant differences between species, but not habitat or diet, in amino acid metabolism, energy metabolism, and metabolism of terpenoids and polyketides.

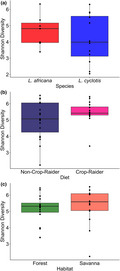

## 1 INTRODUCTION

The animal gut is generally comprised of trillions of microorganisms collectively known as the gut microbiome. In some species, the gut microbiome has been described as an evolved mutualism in which microorganisms assist their hosts with ecological interactions, immunity, nutrient uptake, energy acquisition, and digestion of materials that would otherwise be impossible (Russell, Dubilier, & Rudgers, 2014; Stevens & Hume, 1998; Vavre & Kremer, 2014). However, this is not a universal phenomenon, as some animal taxa do not appear to exhibit microbial dependency. Studies have shown that some invertebrates (Lucarotti, Whittome‐Waygood, & Levin, 2011; Shelomi, Lo, Kimsey, & Kuo, 2013; Whittome, Graham, & Levin, 2007), and specifically caterpillars (Hammer, Janzen, Hallwachs, Jaffe, & Fierer, 2017), lack a definitive resident microbiome. Additionally, Hammer et al. (2017) provided evidence of low bacterial abundance in some vertebrates, including the brant goose *Branta bernicla* and the little brown bat *Myotis lucifugus*. Given the complexity of these systems and the vast diversity of species, it is important to understand the factors that influence the composition of microbial communities and the roles those communities play in the evolution of species.

For species that have a resident microbiome, diet is considered one of the primary factors influencing the diversity and composition of the microbial community (Muegge et al., 2011). Different food substrates promote growth of microbial taxa with specialized metabolic functions, which can lead to variation in taxonomic abundance (De Filippo et al., 2010; Scott, Gratz, Sheridan, Flint, & Duncan, 2013; Wang et al., 2013). For example, drastic changes in diet that occur across life stages cause concomitant shifts in the gut microbiome (Ilmberger et al., 2014; Kohl, Cary, Karasov, & Dearing, 2013). Similarly, dietary differences among European and African human populations drive the evolution of microbiomes containing unique species (De Filippo et al., 2010). It has also been shown that habitat can have a considerable influence on the gut microbiome (Amato et al., 2013); however, this effect is most likely explained by the association between habitat and the food resources available (Barelli et al., 2015).

In addition to diet, host phylogeny has been found to influence the microbiome, often through genes associated with the immune system (Blekhman et al., 2015; McKnite et al., 2012; Zhang et al., 2010; Zhao et al., 2013). This leads to significant differences in the bacterial community structure among broad groups of animals with varying dietary strategies: herbivores, carnivores, and omnivores (Ley et al., 2008; Nishida & Ochman, 2017). Because diet and phylogeny are often interrelated (Hale et al., 2018), their individual effects may be complex and highly confounded. Microbiome composition may be very similar among some dietary specialists despite their distant phylogenetic relationships, such as anteaters, aardvarks, and aardwolves (Delsuc et al., 2014). Conversely, some closely related species may possess common microbiota even though they differ substantially in diet (Delsuc et al., 2014).

Our understanding of the gut microbiome's response to diet, habitat, and host phylogeny has been primarily derived from controlled experiments on humans (Turnbaugh et al., 2009) and other model species (Murphy et al., 2010). Despite the benefit of experimental manipulation in model organisms, animals that have been reared entirely in sterile laboratory environments are not representative of their wild counterparts (i.e., laboratory mice; Abolins et al., 2017; Beura et al., 2016; Leung, Budischak, The, Hansen, & Bowcutt, 2018; Reese et al., 2016; Rosshart et al., 2017). Thus, the results of controlled experiments cannot always be directly applied to ecological understanding or conservation. The literature on host–microbiome interactions in wild taxa has grown over the last decade (Delsuc et al., 2014), with foci ranging from the influence of ecosystem factors on the gut microbial composition of Neotropical primates (Amato et al., 2016) to the role of local environmental exposures in shaping the bacterial communities of Galápagos iguanas (Lankau, Hong, & Mackie, 2012). However, our knowledge of the community composition of host‐associated microbiomes and the mechanisms driving variation among free‐ranging individuals, populations, and species is still limited (Amato et al., 2016).

The two sister species of African elephant, the African savanna elephant (*Loxodonta africana*) and the African forest elephant (*L. cyclotis*), represent a unique system in which to study the influences of phylogeny, diet, and habitat on microbial communities in wild populations. These species diverged from a common ancestor 2.6–5.6 mya (Rohland et al., 2010) and are the two most closely related taxa within the extant Proboscidea. Although they are closely related and have been found to hybridize in regions of range overlap (Clemens & Maloiy, 1982), they have developed distinguishing ecological and morphometric differences. *L. cyclotis* have substantially smaller body sizes than *L. africana*, their ears are more oval‐shaped, and their tusks point downward unlike the outward curved tusks of *L. africana* (Short, 1981), likely as an adaptation to traversing their thickly forested habitat. The diet of *L. cyclotis* is primarily made up of fruits, leaves, and bark of forest trees (Short, 1981), whereas the diet of *L. africana* consists primarily of grasses and woody browse (Codron et al., 2011). Differences in morphology and dietary variation suggest that there may be disparities in metabolic demands between the two species and that they may utilize unique communities of gut microbes to aid in digestion.

Although *L. africana* and *L. cyclotis* are generally associated with savanna and forest ecosystems, respectively, each species has been observed living in environments more typical of the other. This generality in habitat use can be partly explained by migratory behavior (Galanti, Preatoni, Martinoli, Wauters, & Tosi, 2006), but it is more often attributed to the continued expansion and encroachment of human populations, which force wild elephants into competition for resources (Balmford et al., 2001; Barnes, 1996; Galanti et al., 2006; Hoare, 2000; Hoare & du Toit, 1999; Naughton‐Treves, 1998; Pimm, Russell, Gittleman, & Brooks, 1995; Woodroffe & Ginsberg, 1998). Reported incidences of crop‐raiding, the consumption of human‐grown grains, fruits, and vegetables, are on the rise as human populations expand, and natural habitat is converted to agriculture, largely for the purpose of farming cash crops such as corn (Rode, Chiyo, Chapman, & McDowell, 2006; Sitati, Walpole, Smith, & Leader‐Williams, 2003). Chiyo and Cochrane (2005) found that *L. africana* crop‐raiders obtained up to 38% of their daily forage from agricultural crops. In general, elephants are thought to resort to crop‐raiding due to increasing proximity of their natural habitat to agricultural land, anthropogenic degradation of their food resources, and greater palatability and nutritional value of cultivated plants (Sach, Dierenfeld, Langley‐Evans, Watts, & Yon, 2019; Sukumar, 1990). Additionally, Finch (2013) suggested that crop‐raiding behavior may be associated with decreased parasitic loads in *L. africana*. Despite the potential ecological benefits to elephants, crop‐raiding is a high‐risk behavior, as the destruction of property can cost the elephants their lives. With increased pressure from human encroachment, human–elephant conflict is at the forefront of management concerns, so it is necessary to understand the environmental and physiological factors impacting the ecology and evolution of the African elephants.

In this study, we examined the effect of host phylogeny as well as diet, through habitat‐based diet type and crop‐raiding, on the microbiome of free‐ranging *L. africana* and *L. cyclotis*. By comparing sequences of the 16S rRNA gene amplified from African elephant fecal samples, we compared the diversity, taxonomy, and predicted metabolic function of microbial communities between the two elephant species. We then tested for differences between crop‐raiding and noncrop‐raiding individuals from both savanna and forest habitats within *L. africana*. We hypothesized that variation in habitat type and crop‐raiding behavior between populations and individuals of *L. africana* would result in significant differences in their microbiota. Additionally, we predicted that *L. cyclotis* would have a more diverse microbiome as a result of living in the highly diverse tropical forests of Central Africa.

## 2 MATERIALS AND METHODS

### 2.1 Study sites and sample collection

**FIGURE 1:**
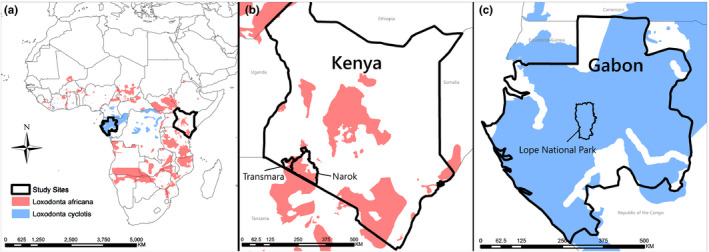
Locations of the study areas including *Loxodonta africana* (red) and *L. cyclotis* (blue) distributions across Africa (a); the Narok and Transmara Districts surrounding Maasai Mara National Reserve in Kenya (b); and Lope National Park in Gabon.

We collected approximately 20 g of fecal sample from elephant dung piles within 12 hr of excretion (based on sample moisture and odor) in Kenya and Gabon, in areas where historical or contemporary genetic hybridization between *L. africana* and *L. cyclotis* was unlikely (Figure 1a). All samples were collected during the wet season at all sites to minimize the effect of environmental variability on sample quality. Using a plastic tape measure, we also took up to three bolus circumference measurements from sampled dung piles to serve as a proxy for age using the standards established by Morrison, Chiyo, Moss, and Alberts (2005) for *L. africana* and Schuttler, Whittaker, Jeffery, and Eggert (2014) for *L. cyclotis*. In compliance with requirements under USDA‐APHIS permit #128686, the tubes containing samples were boiled to prevent the transmission of pathogens. They were stored in Queen's College Preservation Buffer (20% DMSO, 0.25 M EDTA, 100 mM Tris, pH 7.5, saturated with NaCl; Amos, Whitehead, Ferrari, Payne, & Gordon, 1992) at room temperature in the field and exported to the United States where they were stored at −20°C prior to DNA extraction. In a comparison of storage methods for fecal samples, Kawada, Naito, Andoh, Ozeki, and Inoue (2019) found that the fecal microbiota detected in samples stored in this buffer clustered with fresh samples in a PCoA and found no effect of storage buffer on alpha or beta diversity.

*Loxodonta africana* samples were collected during a single field season between May and July 2011 (Figure 1b). Samples collected from the Transmara District of southwestern Kenya (*n* = 20) were categorized as forest habitat as this region is characterized by semideciduous, dry deciduous, and acacia woodlands (Sitati et al., 2003). Samples collected from the Loita Plains of the Narok District (*n* = 15) were classified as savanna habitat as the area is characterized by dwarf shrub and whistling thorn (*Acacia drepanoligium*) grasslands (Serneels & Lambin, 2001). In addition, we collected samples from crop‐raiding events to compare the effects of agricultural products (primarily maize) and natural vegetation on the gut microbiome. Our field team was notified the morning after crop‐raiding events by a World Wide Fund for Nature scout team who worked with local farmers on crop‐raiding issues. On the same day as the reported incident, we collected elephant fecal samples from raided fields and classified them according to surrounding natural habitat type to allow comparative analyses. All *L. africana* samples were classified by habitat and diet type: savanna diet (*Laf*:S, *n* = 12), savanna diet with crop‐raiding (*Laf*:S + CR, *n* = 3), forest diet (*Laf*:F, *n* = 7), and forest diet with crop‐raiding (*Laf*:F + CR, *n* = 13).

Samples of *L. cyclotis* (*n* = 13) were collected at Lope National Park, Gabon (Figure 1c), between March and May 2010. This area is characterized by a mosaic of diverse dense tropical forest types with small open patches that served as observation areas in a previous study of forest elephant social structure (Schuttler, Philbrick, Jeffery, & Eggert, 2014). *L. cyclotis* samples were collected within 12 hr of observation from elephants that were not known to raid crops.

While we were able to collect samples from different habitat types within *L. africana,* only forest individuals without known access to agricultural crops were sampled for *L. cyclotis.* Therefore, we were unable to combine all samples for downstream analyses due to the potential confounding effects of phylogeny, habitat, and presence of crop‐raiding. To counteract this problem, we divided all samples into two subsets for all analyses. Subset A contained all *L. africana* habitat and diet types: savanna (*Laf*:S), savanna with crop‐raiding (*Laf*:S + CR), forest (*Laf*:F), and forest with crop‐raiding (*Laf*:F + CR), allowing us to assess the effects of diet, specifically by habitat type and the presence of agricultural products in the diet, while controlling for the possible effect of phylogeny. Subset B contained only individuals from forested habitat with no known addition of agricultural products from *L. africana* (*Laf*:F) and *L. cyclotis* (*Lcy*:F), to allow us to accurately assess the effect of phylogeny while eliminating the potential confounding effects of habitat and diet. We used Subset A for all diet comparisons with habitat and crop‐raiding, and Subset B for all phylogeny comparisons between *L. africana* and *L. cyclotis.*

To prevent sequencing multiple fecal samples from the same elephant or close relatives, *L. africana* and *L. cyclotis* samples underwent DNA extraction using the Qiagen QIAamp DNA Stool Mini Kit (Qiagen) with the modifications described in Archie, Moss, and Alberts (2003) that include increased volume of starting material as well as increased proteinase K volume and digestion time to maximize the final DNA concentration. They were then genotyped at 10 (*L. africana*) and 12 (*L. cyclotis*) microsatellite loci (Finch, 2013; Schuttler, Whittaker, et al., 2014). To determine the sex of each genotyped sample, we amplified two Y‐specific fragments (*SRY1* and *AMELY2*) and one X‐specific fragment (*PLP1*), a sexing technique described by Ahlering, Hailer, Roberts, and Foley (2011). For all extractions and PCRs, we used both negative controls to detect contamination of the reagents and positive controls to standardize allele scoring. We computed pairwise relatedness between samples using ML‐RELATE (Kalinowski, Wagner, & Taper, 2006); when comparisons yielded a coefficient of relatedness >0.25, one of the two samples was omitted. Exception was given to subadult and adult males that were sampled in different habitats than related females, as males are the dispersing sex. Subadult and adult males found in different habitats were assumed to have dispersed; after dispersal, we assumed they were making dietary choices independent of their family groups. For each sample, sex and age of elephant were determined as a possible variable for microbiome differentiation.

### 2.2 Microbial extraction and sequencing

For microbial DNA extraction, we used bead beating (Yu & Morrison, 2004) with modification to accommodate double the starting material (0.50 g). From each sample, the hypervariable V4 region (253 bp) of the bacterial and archaea 16S rRNA gene, recommended by Liu, Chen, Wang, Oh, and Zhao (2005), was amplified using PCR forward primer 515F (5′‐GTG CCA GCM GCC GCG GTA‐A3′) and reverse primer 806R (5′‐GGA CTA CHV GGG TWT CTA AT‐3′; Caporaso et al., 2010; Gilbert et al., 2010). We modified each primer to include Illumina forward, reverse, and multiplex sequencing primers and added a unique 6 bp barcode to each reverse primer (Bartram, Lynch, Stearns, Moreno‐Hagelsieb, & Neufeld, 2011) using the TruGrade service to reduce the risk of oligo crosstalk and barcode misalignment during downstream applications (IDT). PCR amplification was carried out in triplicate, 50 µl reactions containing the following: 1× PCR gold buffer, 0.2 µM dNTPs, 0.5 U AmpliTaq Gold DNA Polymerase (Applied Biosystems), 1.5 mM MgCl_2_, 10× BSA (New England Biolabs), 0.4 µM forward primer, 0.4 µM reverse primer, and 2 µl DNA template. Reactions consisted of denaturation at 95°C for 5 min; 35 cycles of 95°C for 1 min, 50°C for 1 min, and 72°C for 1 min; and 72°C for 10 min. Amplicons were visualized in a 2% agarose gel, purified using an AxyPrep Mag PCR clean‐up kit (Axygen), quantified using a Fragment Analyzer (Advanced Analytical), and combined in equimolar concentrations. Paired‐end 250 nucleotide multiplex sequencing was performed for the resulting libraries using Illumina MiSeq at the University of Missouri DNA Core Facility.

### 2.3 Microbial bioinformatic pipeline

Raw sequencing reads were processed using qiime 2 v.2019.1 (Caporaso et al., 2010). Metadata files were verified for formatting using keemei (Rideout et al., 2016). Paired‐end data were joined using vsearch (Rognes, Flouri, Nichols, Quince, & Mahé, 2016), and quality scores filtered within quality‐filter (Bokulich et al., 2013) plugins using default parameters. Joined sequences and their corresponding quality scores were visually inspected on the qiime2 interactive viewer to obtain optimal trim length. Sequences were filtered using these quality scores, singletons and chimeras were removed, and resulting sequences were length trimmed with the plugin deblur (Amir et al., 2017) using its denoise‐16S positive filter workflow to collapse into 99% similar operational taxonomic units (OTUs). Reads were taxonomically classified with sklearn (Fabian et al., 2011) using a Native Bayes classifier first trained on the 99% OTU 515F/806R region sequences from the GreenGenes database (DeSantis et al., 2006). Raw microbial OTU abundances were imported from qiime 2 into *r* v. 3.5.2 (R Core Team, 2018) and rarefied to an even sampling depth using the package *qiime2r v.* 0.99.11 (Bisanz, 2018).

### 2.4 Core microbiome characterization

To identify the taxonomic level that was most appropriate to characterize the core microbiome, we calculated the proportion of unique OTUs that were successfully classified at the previously described 99% certainty to any given taxonomic level (kingdom, phylum, class, order, family, genus, or species). We then compared the proportion of classified OTUs at each taxonomic level with average confidence to determine the optimal taxonomic level at which to characterize the microbiome of all samples, which we defined as the most specific taxonomic level that maintained a high average confidence of classification.

We characterized the core microbiome based on habitat, presence or absence of crop‐raiding, and phylogeny as the classified OTUs present in 100% of respective elephant samples and then summarized them at their previously identified taxonomic level (Hamady & Knight, 2009; Shade & Handelsman, 2012; Turnbaugh et al., 2007). Only those classified OTUS with an average relative abundance greater than 10% across samples were considered for comparison. Rarefied abundance data were square root‐transformed and analyzed with general linear mixed models (GLMM) assuming a negative binomial distribution using the *glmer.nb* function in the *R* package lme4 *v.* 1.1‐21 (Bates, Maechler, Bolker, & Walker, 2015), with elephant sex included in each model as a random effect. We did not analyze data with respect to elephant age at the species level. Although we were able to obtain juveniles, subadults, and adults for *L. africana*, we were only able to obtain samples from adults for *L. cyclotis*. However, preliminary analyses revealed that within *L. africana,* alpha diversity (GLMM; *p* = .885) and beta diversity (PERMANOVA; *p* = .126) did not differ significantly among age groups. Therefore, age was not included as a random effect in any downstream analyses.

We extracted *p*‐values from all negative binomial GLMM with the ANOVA function using the *R* package car *v*. 3.0‐3 (Fox & Weisberg, 2011) and corrected *p*‐values for false discovery rate (FDR). For models showing significant differences among treatments (habitat, diet, and phylogeny), we ran post hoc Tukey tests on all pairwise comparisons using the *R* package multcomp *v*. 1.4‐10 (Hothorn, Bretz, & Westfall, 2008). For those showing significant differences in microbial abundance, we further tested for differences at lower taxonomic levels, considering only genera with an average relative abundance >10% and using GLMM with a negative binomial distribution.

### 2.5 Diversity analyses

We calculated alpha diversity as the Shannon diversity index, in vegan *v.* 2.5.4 (Oksanen, Blanchet, Friendly, Kindt, & Legendre, 2017) for habitat and diet (Subset A) and phylogeny (Subset B). We compared average Shannon Diversity values among habitat, diet, and phylogeny treatments using GLMM in the package Lme4, including elephant sex in each model as a random effect. For analysis of diet and habitat, we first tested for the presence of a diet–habitat interaction before assessing main effects in a separate GLMM.

We evaluated beta diversity between diets using data Subset A and phylogeny using Subset B by calculating Bray–Curtis dissimilarity and running 9999 permutations in PERMANOVA (Anderson, 2001) vegan v. 2.5.4 (Oksanen et al., 2017). For all comparisons, we included sex as a stratum that is akin to setting a random effect in a linear mixed model; for diet and habitat, we first tested for a diet–habitat interaction before assessing main effects in a separate PERMANOVA. We visualized beta diversity with nonparametric multidimensional scaling (NMDS) in vegan 2.5.4. Finally, we assessed differences in within‐population variability between diet and phylogeny using the centroid method of the beta dispersion test in vegan 2.5.4.

### 2.6 Functional analysis

Unique OTUs underwent metagenome prediction steps using the custom‐tree‐pipeline in picrust2 (Langille et al., 2013) using the plugin q2‐picrust2. To obtain metabolic pathway abundance, we referenced the picrust output using the Kyoto Encyclopedia of Genes and Genome (KEGG) Orthology (KO) (Kanehisa & Goto, 2000; Kanehisa et al., 2014). Pathways were then clustered by metabolism using the BRITE function hierarchies within KEGG, representing higher‐order metabolic functions. We tested for significant differences in mean metabolic contribution at KEGG Level B (based on BRITE hierarchy) between African elephant species, habitat, and diet and treatments using GLMM and correcting for FDR. Metabolic contribution was calculated by dividing raw pathway abundance by the sum total abundance for all pathways within an individual sample. It is important to note that picrust analyses, along with all predictive functional gene analysis programs (i.e., tax4fun, Abauer, Wemheuer, Daniel, & Meinicke, 2015; cowpi, Wilkinson et al., 2018; humann2, Abubucker et al., 2012), should always be considered speculative, as predicted microbial genes may not always reflect accurate metagenomic processes. Here, we use KEGG functional predictions as an additional comparison method to potentially explain differences between our elephant treatments and provide a starting point for future metabolome studies rather than an exact illustration of metabolome processes occurring in the African elephants.

## 3 RESULTS

### 3.1 Sequencing results

Sequencing of the 16S rRNA gene from these 48 samples produced 23,674,488 reads with a mean of 493,218.5 reads per sample (median 255,112.5; minimum 10,639; maximum 11,395,868). Following joining of paired reads with vsearch, we obtained 20,242,213 joined reads with a mean of 421,712.8 reads per sample (median 217,426.5; minimum 8,252; maximum 9,835,668). Using the Interactive Quality Plot viewer, we determined that quality of reads began deteriorating at position 252 with 75% of 10,000 randomly resampled forward reads without replacement assigning to this length or less with sequence median PHRED quality score of 39 (low 34; high 41). Deblur filtering, dereplication, and chimera removal resulted in a mean of 141,563 reads per sample (minimum 271; maximum 3,586,017) with a mean of 4,401 unique reads per sample. Unique reads were collapsed at 99% similarity into 9,066 operational taxonomic units (OTUs).

For downstream analyses, we rarefied the full abundance dataset evenly to 8,248 unique OTUs with 11,460 reads per sample after removal of a single sample (OB182; *L. cyclotis*) due to low read count. We assessed the microbiome of all five (four *L. africana* diet treatments and one *L. cyclotis*) African elephant treatments (*n* = 47) at the level of order based on the correspondence between mean confidence and proportion of classified taxa at this level.

### 3.2 Fecal microbiome composition

Of classified OTUs, 100% were classified to the kingdom level, 97.93% to phylum, 97.51% to class, 96.03% to order, 67.70% to family, 25.14% to genus, and 2.99% to species, with 96.15 classification confidence overall (Figure S1). Thus, the lowest taxonomic group at which mean confidence and proportion of successfully classified OTUs coincided was the level of order, with mean confidence of microbial classification at approximately 96.31% (Figure S1). Therefore, the core microbiome was assessed at the level of order. For the characterization of the microbiome, we removed one *L. cyclotis* individual (M10) from the evaluation. While this sample had average microbial read counts, it had a drastically different community composition than all other *L. cyclotis* or *L. africana* individuals. Inclusion of this individual in the determination of the core microbiome resulted in a substantial reduction of bacterial orders for *L. cyclotis.* This individual was, however, retained for diversity analyses.

We found 16 microbial phyla across all samples, and the African elephant treatments varied in the proportion of OTUs assigned to each bacterial phylum (Table S1).The microbiome of all *L. africana*, regardless of diet or habitat type, was dominated by Firmicutes (~40%) with both Bacteriodetes (~20%) and Proteobacteria (~20%) as important contributors (Table S1). In contrast, the microbiome of *L. cyclotis* was dominated by Proteobacteria (~52%), with lower proportions of Firmicutes (~17%) and Bacteroidetes (~14%).

Within all phyla, we found a total of 58 microbial orders at varying proportions within African elephants; however, only 18 of these orders were shared by all individuals (Table S2). An additional six orders were shared at 100% frequency among *L. cyclotis* individuals, and two additional orders were shared among all *L. africana* individuals (Table S2). When evaluating the effect of diet, 17 orders were shared between crop‐raiders and noncrop‐raiders. While four additional bacterial orders were shared among all crop‐raiders, no additional orders were shared among all noncrop‐raiders (Table S2). When evaluated by habitat type, there were 17 shared microbial orders, and while savanna habitat had another six orders found in all individuals, forest habitat had none (Table S2).

**FIGURE 2:**
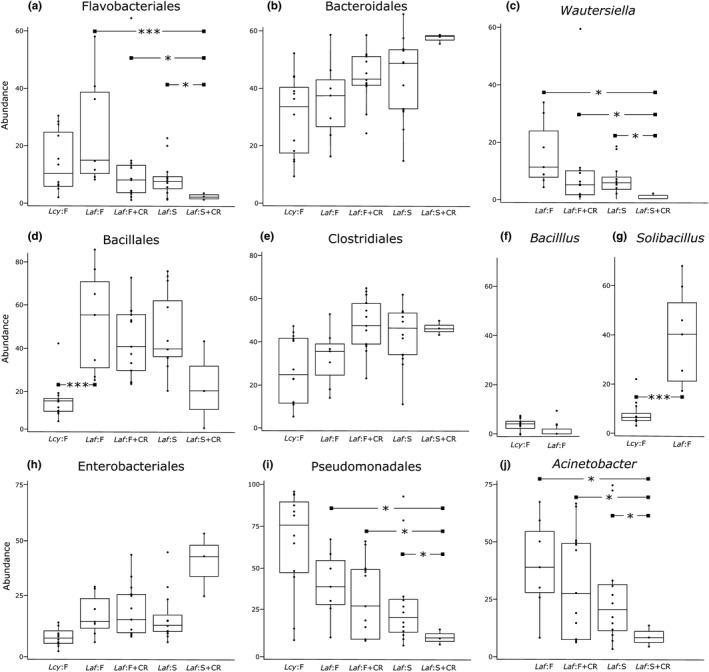
Abundance for the most abundant bacterial orders and genera that differed significantly within elephant treatments: *Loxodonta cyclotis* (*Lcy*:F), *L. africana* forest diet (*Laf*:F), *L. africana* forest diet with crop‐raiding (*Laf*:F + CR), *L. africana* savanna diet (*Laf*:S), and *L. africana* savanna diet with crop‐raiding (*Laf:*S + CR). Within the three most abundant bacterial phyla: Bacteroidetes (a–c), Firmicutes (d–g), and Proteobacteria (h–j), we examined differences at the order level (a, b, e, f, h, i) and genus level (c, d, g, j). (*p < .05, ***p ≤ .001)

The most abundant orders were assessed within the three phyla with the highest average relative abundance across all samples: Bacteroidetes (19.37%), Firmicutes (37.82%), and Proteobacteria (27.92%). Within Bacteroidetes, the orders Flavobacteriales (Figure 2a) and Bacteroidales (Figure 2b) accounted for the highest average number of microbial OTUs. While neither order was significantly different between elephant species, Flavobacteriales differed significantly among diet treatments (GLMM; *F* = 8.268, *p* = .0046), with significant post hoc differences between *Laf*:S + CR and *Laf*:F + CR (Tukey test; *p* = .005), *Laf*:F and *Laf*:S + CR (*p* = .0001), and *Laf*:S and *Laf*:S + CR (*p* = .014; Figure 2a). Within Flavobacteriale*s*, the genus *Wautersiella* accounted for the most OTU abundance and differed significantly among diet types (*F* = 6.976, *p* = .003, Figure 2c), with significant post hoc differences between *Laf:*S + CR and *Laf*:F + CR (*p* = .002), *Laf*:F and *Laf*:S + CR (*p* = .002), and *Laf*:S and *Laf*:S + CR (*p* = .016).

Within Firmicutes, the orders Bacillales (Figure 2d) and Clostridiales (Figure 2e) accounted for the most microbial OTUs, and Bacillales differed significantly between elephant species (*F = *30.623, *p* < .001). Within Firmicutes: Bacillales, the genera *Bacillus* (Figure 2f) and *Solibacillus* (Figure 2g) constituted most of the OTU abundance, and *Solibacillus* differed significantly between *L. africana* and *L. cyclotis* (GLMM; *F = *42.547, *p < *.001).

Within Proteobacteria, the orders Enterobacteriales (Figure 2h) and Pseudomonadales (Figure 2i) accounted for the most microbial OTUs. Neither were significantly different between species, but Pseudomonales differed significantly among diet types (*p* = .005), with significant post hoc differences between *Laf*:S + CR and *Laf*:F + CR (Tukey test; *p* = .0029), *Laf*:F and *Laf*:S + CR (*p* = .0024), and *Laf*:S and *Laf*:S + CR (*p* = .0026; Figure 2i). Within Proteobacteria: Pseudomonadales, the genus *Acinetobacter* accounted for the most OTU abundance and differed significantly among diet types (GLMM; *F = *4.760, *p* = .003), with significant post hoc differences between *Laf:*S + CR and *Laf*:F + CR (Tukey; *p* = .003), *Laf*:F and *Laf*:S + CR (*p* = .003), *Laf*:S and *Laf*:S + CR (*p* = .002; Figure 2j).

### 3.3 Alpha diversity

Shannon diversity for all samples ranged from 2.047 to 6.500, where the average for *L. africana* was 4.681 ± 0.996 and the average for *L. cyclotis* was 4.186 ± 1.49. Shannon diversity did not differ significantly between species (GLMM; *p* = .449; Figure S2A). In our two‐way mixed‐effect models for diet types, we found no significant interaction between habitat and diet types (GLMM; *p* = .757). Within diet types, the effects of crop‐raiding (GLMM; *p* = .126; Figure S2B) and habitat (*p* = .239; Figure S2C) were also not significant.

### 3.4 Beta diversity

**FIGURE 3:**
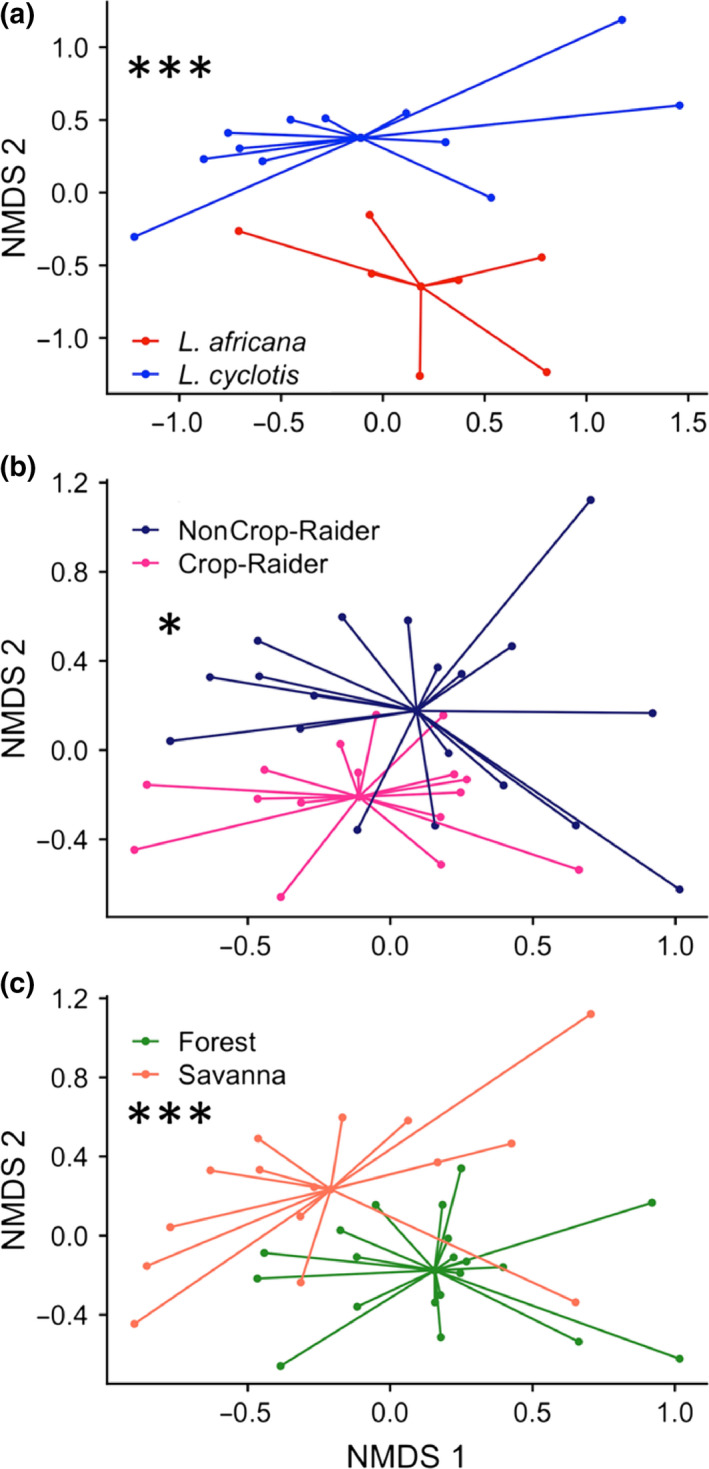
Nonparametric multidimensional scaling (NMDS) showing beta diversity based on Bray–Curtis distances for rarefied OTU abundance by (a) African elephant species, (b) diet type, and (c) habitat. (*p < .05, ***p ≤ .001)

We found significant differences in beta diversity between species (PERMANOVA; *p* = .001; Figure 3a). Within diet types, the effects of crop‐raiding (*p* = .007; Figure 3b) and habitat (*p* < .001; Figure 3c) were also significantly different between groups within *L. africana.* We detected no significant interactions between diet and habitat (PERMANOVA; *p* = .099). Beta dispersion was not different between species (Beta Dispersion; *F* = 0.64, *p* = .435), diet type (*F* = 2.514, *p* = .122), or habitat (*F* = 2.827, *p* = .102).

### 3.5 Functional analyses

**FIGURE 4:**
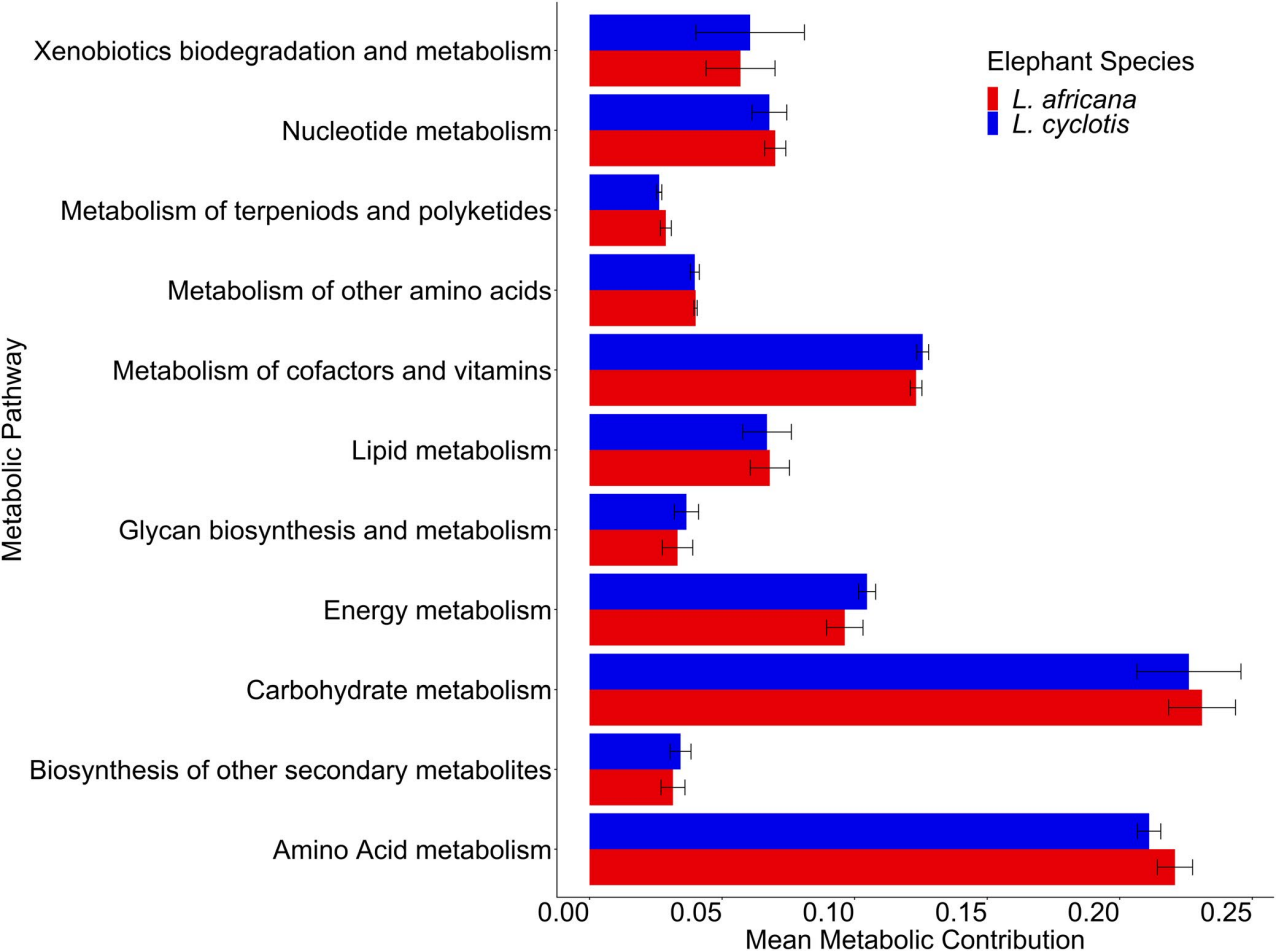
Mean metabolic contribution, calculated as the proportion of an individual sample's metabolic profile comprised by a given pathway, and standard deviation of KEGG metabolic pathways that were significantly different between African elephant species. The primary function of the represented pathway is listed on the *x*‐axis. (*p < .05)

picrust2 determined 6,906 KEGG pathways values from which we clustered into a corresponding BRITE hierarchy representing 11 higher‐order metabolic functions (Level B) and the 155 lower‐order metabolic functions that comprised them (Level C). Metabolism of terpenoids and polyketides (GLMM; *p* = .006), energy metabolism (*p* = .006), and amino acid metabolism (*p* = .006) were all significantly different between species (Figure 4). Metabolism of cofactors and vitamins also trended toward significance (*p* = .075) between species. No predicted metabolic functional pathways were significantly different between habitats or diets.

## 4 DISCUSSION

### 4.1 The elephant microbiome

The core microbiome among mammalian lineages varies a great deal in the relative abundance of each microbial phylum; however, across mammalian taxa, four bacterial phyla appear to be key drivers: *Actinobacteria*, *Bacteroidetes*, *Firmicutes*, and *Proteobacteria* (Nishida & Ochman, 2017). While *Bacteroidetes*, *Firmicutes,* and *Proteobacteria* are the dominating phyla in African elephants, *Actinobacteria* were in low abundance in both species; instead, *Verrucomicrobia* comprised the next largest proportion of the elephant's microbiome, regardless of diet or habitat.

We found that the microbiome of all *L. africana*, regardless of diet or habitat type, was dominated by *Firmicutes*, but that both *Bacteriodetes* and *Proteobacteria* were important contributors. This ratio of high *Firmicutes* (~40%) to lower *Bacteroidetes* (~20%) and *Proteobacteria* (~20%) is most comparable to other hindgut‐fermenting species such as Asian elephants (*Elephas maximus*, Ilmberger et al., 2014), horses (*Equus caballus;* O'Donnell et al., 2013; Proudman et al., 2015), black rhinoceros (*Diceros bicornis;* Antwis, Edwards, Unwin, Walker, & Schultz, 2019), white rhinoceros (*Ceratotherium simum*; Bian, Ma, Su, & Zhu, 2013), rabbits (*Oryctolagus cuniculus*; Eshar & Weese, 2014), and even the distantly related koala (*Phascolarctos cinereus;* Barker, Gillet, Polkinghorne, & Timms, 2013), demonstrating a strong correlation with gut physiology rather than phylogeny. Our results are additionally supported within broad‐scale microbiome studies where *L. africana* are used during phylogeny comparisons (Ley et al., 2008; Muegge et al., 2011) with elephants clustering with other hindgut‐fermenting herbivores.

To our knowledge, our study is the first to investigate the microbiome of *L. cyclotis.* In contrast with the microbiome of *L. africana*, we found that the microbiome of *L. cyclotis* was dominated by *Proteobacteria* (~52%), with lower proportions of *Firmicutes* (~17%) and *Bacteroidetes* (~14%). This shift in dominating bacterial phyla to *Proteobacteria* is most comparable to certain *Phyllostomid* bats (Carrillo‐Araujo et al., 2015; Ley et al., 2008), gorilla sp. (*Gorilla beringei*, *Gorilla gorilla*; Ley et al., 2008; Moeller et al., 2013), and even frugivorous neotropical birds (García‐Amado et al., 2018). Interestingly, dominating proportions of *Proteobacteria* have been detected in elephants previously, but only in captive juveniles, a three‐week‐old Asian elephant (Ilmberger et al., 2014), and a seven‐month‐old *L. africana* (Ley et al., 2008). However, all of our samples for *L. cyclotis* were from adults, leaving us to hypothesize that this may be a result of the dietary difference in *L. cyclotis,* which is higher in fruit, and therefore has a higher proportion of simple carbohydrates and a lower fiber content (Moermond & Denslow, 1985) than the primarily woody browse and grasses that compose the *L. africana* diet.

Our ability to predict functional differences in the microbial communities of the elephant species was limited by the fact that the KEGG database primarily reflects information gleaned from studies of humans and model organisms. There have been very few studies of the microbiome in wild species, especially those such as *L. cyclotis*, whose habitats are remote and inaccessible. Thus, we are only able to make limited inferences. In our dataset, *L. africana* was significantly higher for metabolism of terpenoids and polyketides (*p* = .006) and amino acid metabolism (*p* = .006). Although structurally diverse, the dominant types of polyketides are widespread as antibiotics, antifungals, and antiparasitics. *L. cyclotis* was significantly higher for energy metabolism (*p* = .006). These functional differences may indicate a difference in energy allocation and metabolic capabilities and illustrate the need for metabolomic comparisons between the species.

### 4.2 Habitat and diet

While both diet and body size can affect alpha diversity in the microbiome of a broad variety of taxa, the most important predictor of alpha diversity has been found to be gut physiology (Reese & Dunn, 2018). Animals with simple guts, such as carnivores, typically have lower microbial richness than foregut ruminants or hindgut fermenters. As large‐bodied hindgut fermenters, African elephants would be expected to have the relatively high alpha diversity we found in this study (Figure S2). We were surprised, however, that the Shannon diversity index revealed no significant differences in the abundance or evenness of microbial taxa between species, diets, and habitats. If microbiome diversity is correlated with diet diversity, African elephants that live in the highly diverse tropical forest environments would be predicted to have higher alpha diversity. Our finding that differences in diet and habitat were not associated with differences in alpha diversity is in agreement with the results of Kartzinel, Hsing, Musili, Brown, and Pringle (2019), who found that in general species with diverse diets did not have the most diverse microbiomes. Taken together, our studies suggest that we are only beginning to learn about the mechanisms that underlie the diversity of the microbiome.

Within *L. africana,* we found overall differences, as reflected by beta diversity, between the microbiota of crop‐raiding and noncrop‐raiding elephants, as well as between individuals that live in savanna and forest habitats. Although there were few statistically significant differences found within microbial orders between groups, there may be biologically significant differences. For instance, one of the secondary metabolites of a species of *Wautersiella*, which differed between *Laf*:S + CR and all other groups, has been found to be active against nematodes in laboratory tests (Chen, Wang, Zhang, & Li, 2015)*.* In addition, savanna elephants that live near human populations encounter different environmental conditions than those that live in less anthropogenically affected habitats. To the extent that the microbiome is involved in the stress response (Maloney, Desbonnet, Clarke, Dinan, & Cryan, 2014), small differences in the encounter rate of different bacterial strains may affect elephants. For instance, the genus *Sporosarcina*, one of the genera within phylum *Firmicutes* that was trending toward significance (GLMM; *p* = .094) between *L. africana* and *L. cyclotis,* includes a species (*S. urease*) that is found in high densities in soils that are subject to animal urine (Pregerson, 1973). The presence of this genus in the microbiome of elephants may reflect the influence of living in close proximity to humans, and livestock, and while speculative, may warrant future study of the indirect effects of proximity of humans and domestic animals on the microbiome of wild species.

Our results, especially in terms of crop‐raiding individuals, may play a role in determining the effect of captivity on the microbiome of elephants. Our study was conducted using field‐collected samples and represents the microbiome of individuals feeding on a variety of wild plant species or agricultural crops. Thus, our study supports previous results that indicate that there are marked differences between the microbiomes of captive and wild individuals (McKenzie et al., 2017; Rosshart et al., 2019), likely as a result of dietary differences (Cheng et al., 2015; Delsuc et al., 2014; Hale et al., 2018; Kong et al., 2014; Zhu, Wu, Dai, Zhang, & Wei, 2011). The single *L. africana* that Nishida and Ochman (2017) included in their study was a captive individual (AfElphSD3, Zoological Society of San Diego), and its microbiome was dominated by *Bacteriodetes*, *Firmicutes,* and *Verrucomicrobia*. While our *L. africana* samples were rich in *Firmicutes* and *Bacteriodetes, Verrucomicrobia* made up only a small fraction of the microbiome. However, crop‐raiding behavior may serve as a proxy for captivity, and in these individuals, we see slightly elevated proportions of *Verrucomicrobia*. Future studies of links between diet and the microbiome may benefit captive populations if researchers are able to establish a diet composition that allows captive elephants to maintain a gut microbiome comparable to that of wild populations.

### 4.3 Implications for species conservation

To our knowledge, this study is the first to characterize differences between the African elephant species and to incorporate the more recently described *L. cyclotis* in a microbiome study. We also examined the effect of diet and habitat on the microbiome. Our results provide a benchmark for understanding the effects of diet and habitat on the microbiome of wild elephants, especially for populations that live in proximity to humans and domestic animals. More extensive microbiome studies into wild populations of Asian elephants have not been conducted but will provide an important and more accurate comparison to this study.

Both *L. africana* and *L. cyclotis* are currently at the forefront of conservation concerns, with *L. africana* often publicized as a member of the charismatic megafauna and an umbrella species to garner additional conservation support for African species. While *L. africana* is already established in captive breeding programs with both the American Association of Zoos and Aquariums (AZA) and European Endangered Species Programme (EESP), *L. cyclotis* currently has no such programs. The significant differences in the microbiomes of the wild African elephant species should be considered if plans are made to attempt to manage *L. cyclotis* in captivity. Current dietary and nutritional recommendations for captive Asian and African elephants are largely extrapolated from the dietary needs of horses (Ullrey, Crissey, & Hintz, 1997), and while our results illustrate that horses may serve as a proxy for *L. africana*, *L. cyclotis* may not be able to utilize nutrients in the same way. This raises concerns for Asian elephant management, as the diet and habitat of that species would be more similar to that of *L. cyclotis.* Although Asian elephants already have established breeding programs, comparisons of captive Asian elephants and horses have shown lower absolute digestibility of the diet for elephants (Clauss, Loehlein, Kienzle, & Wiesner, 2003). To our knowledge, no metagenomic or metabolomic studies have been conducted on wild populations.

As we continue to advance our understanding of host–microbiome associations, we need to increase our focus on how current and new information will aid in the conservation of threatened taxa (Redford, Segre, Salafsky, del Rio, & McAloose, 2012). Studies of humans and model organisms have established links between the microbiome and health, not only through the effects of pathogens but also through alterations in the composition of the microbiota. In this study, we provide evidence that phylogeny, diet, and habitat all independently influence the gut microbiome of the two African elephant species, both of which are considered endangered. Furthermore, the dietary effect we observed in these elephants is in part attributed to crop‐raiding, a behavior that often occurs as a major component of human–elephant conflict. Cultivated crops may be more palatable and, in some cases, more nutritious than analogous wild plants (Sukumar, 1990). As African elephants continue to adapt, both in diet and in habitat tolerance, to expanding human populations, conservation management will be essential to coexistence. For elephants and other wildlife species, conservation management may well depend on gaining a better understanding of the effects of alterations of the microbiome on reproduction and survivorship.

## CONFLICT OF INTEREST

The authors have no conflicts of interest to declare.

## AUTHOR CONTRIBUTION

**Kris Budd:** Data curation (equal); Formal analysis (equal); Visualization (equal); Writing‐original draft (equal); Writing‐review & editing (equal). **Joseph C. Gunn:** Data curation (equal); Formal analysis (equal); Visualization (equal); Writing‐original draft (equal); Writing‐review & editing (equal). **Tabitha Finch:** Conceptualization (equal); Data curation (equal); Investigation (equal); Methodology (equal); Writing‐original draft (equal). **Katy Klymus:** Data curation (equal); Formal analysis (equal); Writing‐original draft (equal); Writing‐review & editing (equal). **Noah Sitati:** Conceptualization (equal); Investigation (equal); Methodology (equal); Project administration (equal); Resources (equal); Writing‐original draft (equal); Writing‐review & editing (equal). **Lori S. Eggert:** Conceptualization (equal); Funding acquisition (equal); Project administration (equal); Resources (equal); Supervision (equal); Writing‐original draft (equal); Writing‐review & editing (equal).

## Supporting information

Fig S1

Fig S2

Table S1

Table S2

## Data Availability

*Data have been archived on publicly accessible repositories:* 16s rRNA Sequence: NCBI/GenBank, https://www.ncbi.nlm.nih.gov/bioproject/PRJNA587772/, Qiime Python Scripts, R Code: https://github.com/Eggert‐Lab/Microbiome
