# Supplementary material for: Effects of diet, habitat, and phylogeny on the fecal microbiome of wild African savanna (Loxodonta africana) and forest elephants (L. cyclotis)
Source: Ecol Evol. 2020 May 18;10(12):5637–50. doi: 10.1002/ece3.6305 (PMC7319146; doi:10.1002/ece3.6305)
Supplement: Supplementary file 1 — Fig S1 [file ECE3-10-5637-s001.docx]

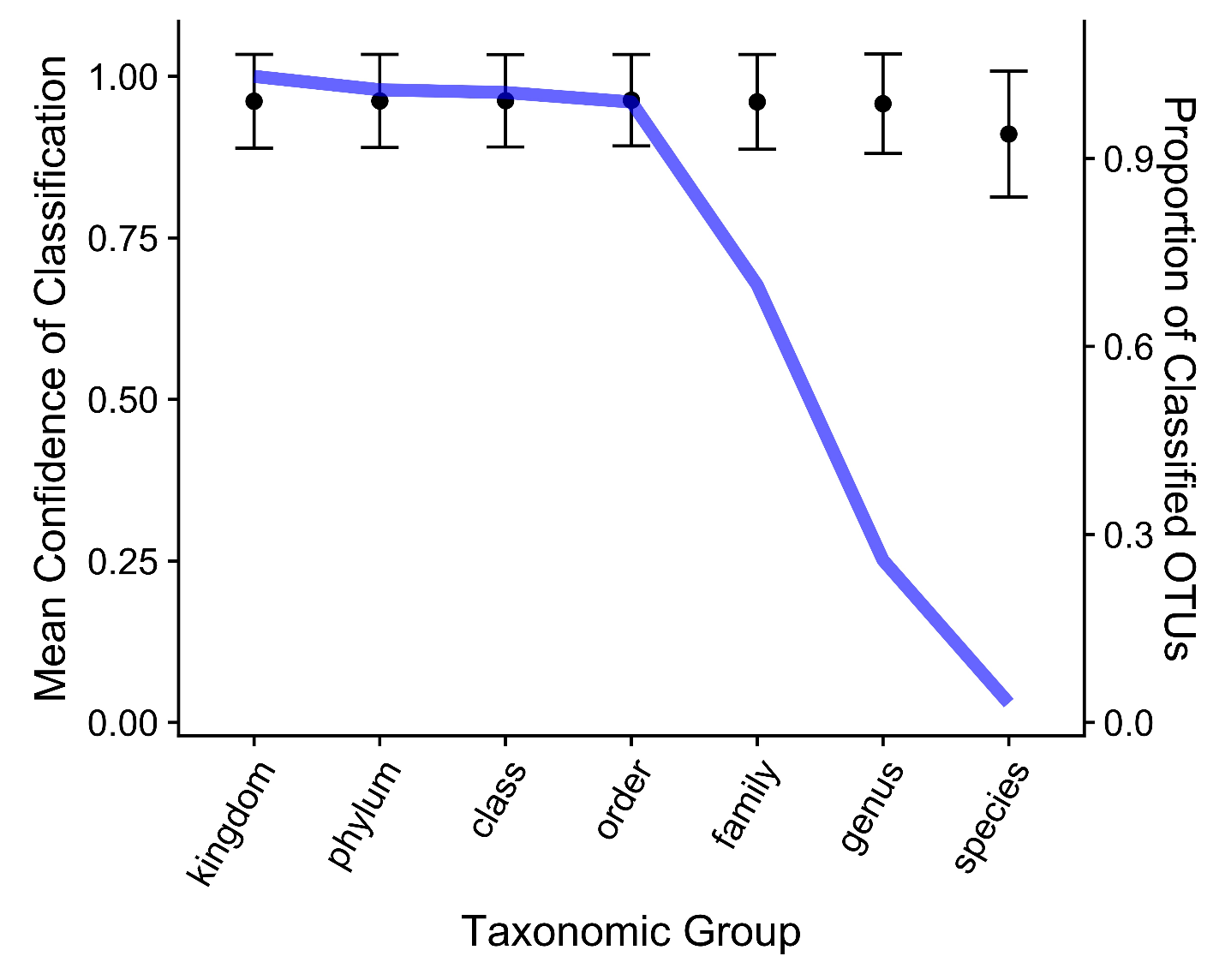


**Supplementary Figure 1:** (*Left axis, black points*) Mean confidence and standard deviation with which microbial OTUs were classified to each taxonomic level before sample rarefaction, and (*right axis, blue line*) proportion of total OTUs successfully classified to each taxonomic group before sample rarefaction.
