# Supplementary material for: Effects of diet, habitat, and phylogeny on the fecal microbiome of wild African savanna (Loxodonta africana) and forest elephants (L. cyclotis)
Source: Ecol Evol. 2020 May 18;10(12):5637–50. doi: 10.1002/ece3.6305 (PMC7319146; doi:10.1002/ece3.6305)
Supplement: Supplementary file 2 — Fig S2 [file ECE3-10-5637-s002.docx]

**
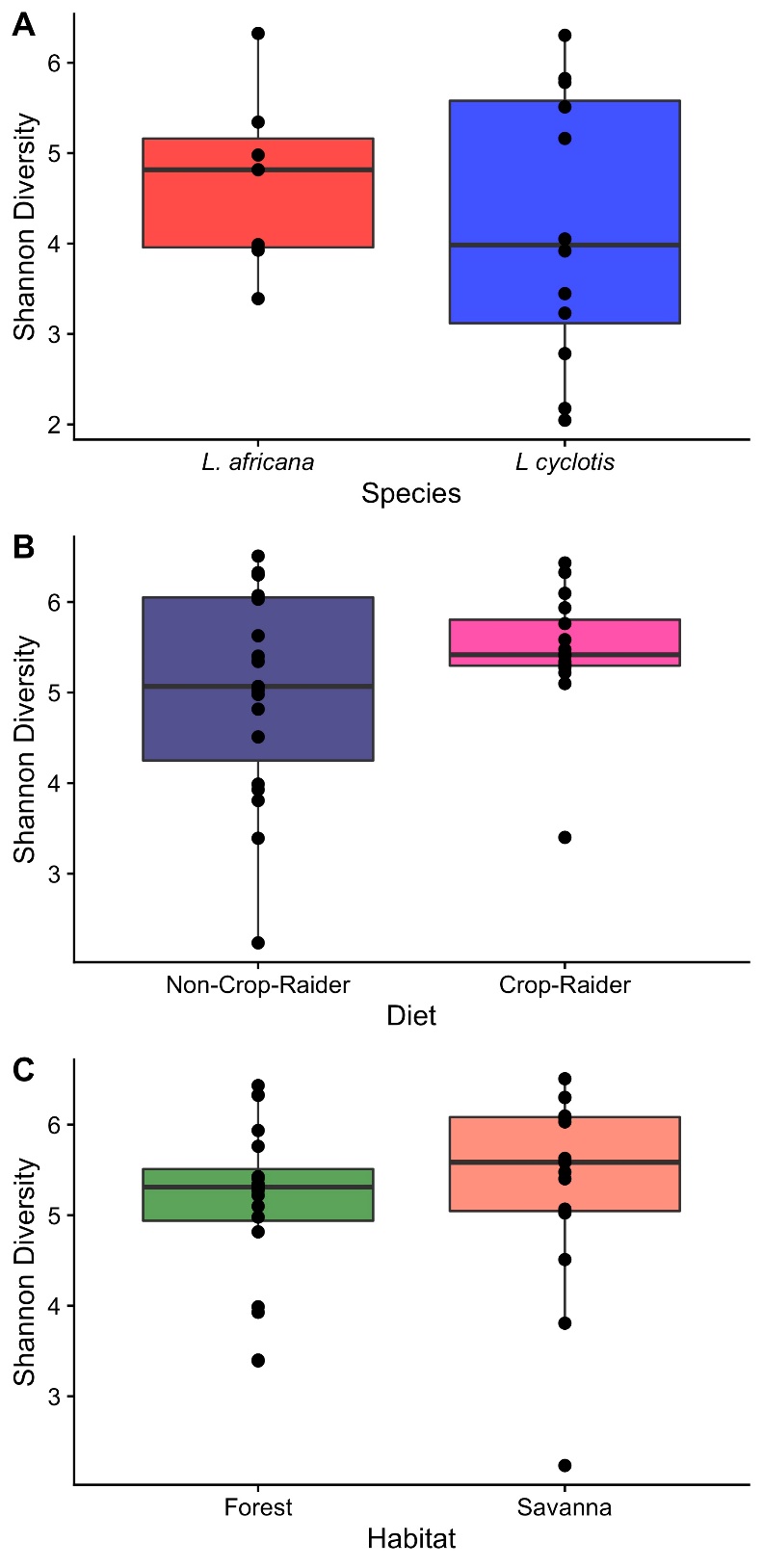
Supplementary** **Figure 2:** Alpha diversity based on the mean of the Shannon Diversity index for rarefied OTU abundance by (A) African elephant species, (B) diet status, and (C) habitat type for *L. africana.* No significance differences in alpha diversity were observed between treatment groups.
